# Evolution of the Percutaneous Coronary Intervention (PCI) and Coronary Artery Bypass Grafting (CABG) Indication and Mortality Rates in Spain from 2010 to 2019

**DOI:** 10.3390/jcdd11110369

**Published:** 2024-11-16

**Authors:** Óscar Gasulla, Antonio Sarría-Santamera, Ferran A. Mazaira-Font, Cielo García-Montero, Oscar Fraile-Martinez, Diego Cantalapiedra, Manuel F. Carrillo-Rodríguez, Belen Gómez-Valcárcel, Miguel Á. Ortega, Melchor Álvarez-Mon, Angel Asúnsolo

**Affiliations:** 1 Hospital Universitari de Bellvitge, Universitat de Barcelona, L’Hospitalet de Llobregat, 08907 Barcelona, Spain; oscargasulla@gmail.com; 2 Department of Surgery, Medical and Social Sciences, Faculty of Medicine and Health Sciences, University of Alcalá, 28801 Alcala de Henares, Spain; manuel.carrillo@uah.es (M.F.C.-R.); belengomval@hotmail.com (B.G.-V.); 3 Department of Biomedical Sciences, Nazarbayev University School of Medicine, Astana 010000, Kazakhstan; 4 Departament d’Econometria, Estadística i Economia Aplicada, Universitat de Barcelona, 08907 Barcelona, Spain; ferranmazaira@gmail.com; 5 Department of Medicine and Medical Specialities, Faculty of Medicine and Health Sciences, University of Alcalá, 28801 Alcala de Henares, Spain; cielo.gmontero@gmail.com (C.G.-M.); oscarfra.7@gmail.com (O.F.-M.); miguel.angel.ortega92@gmail.com (M.Á.O.); mademons@gmail.com (M.Á.-M.); 6 Ramón y Cajal Institute of Sanitary Research (IRYCIS), 28034 Madrid, Spain; 7 Faculty of Medicine, Universidad Europea of Madrid, 28670 Madrid, Spain; diego151099@gmail.com; 8 Networking Research Center on for Liver and Digestive Diseases (CIBEREHD), Immune System Diseases-Rheumatology and Internal Medicine Service, University Hospital Prince of Asturias, 28806 Alcala de Henares, Spain; 9 Department of Epidemiology and Biostatistics, Graduate School of Public Health and Health Policy, University of New York, New York, NY 10027, USA

**Keywords:** coronary intervention, artery disease, mortality, Spain

## Abstract

Percutaneous coronary intervention (PCI) and coronary artery bypass grafting (CABG) are the main interventional treatments for coronary artery disease (CAD) patients. Both procedures are constantly being perfected and developed. This study aims to analyze the evolution of intervention mortality rates of PCI and CABG in recent years in Spain. We use a database of all hospital discharges from CABG and PCI procedures in Spain during two periods, between the years 2010 to 2012 and 2016 to 2019. We elaborate two multivariate regression logistic models to test the differences in mortality between the two periods and the two procedures, adjusting the mortality rates by age, gender, and comorbidities. We find strong evidence that CABG significantly reduced mortality rates, especially in complex patients, while PCI remained almost constant. We also discuss how physicians incorporate the improvement in procedures’ performance into the decision-making for the recommendation of these two procedures in CAD patient management.

## 1. Introduction

Coronary artery disease (CAD) is the leading cause of mortality and loss of disability-adjusted life years (DALYs) worldwide, accounting for nearly 7 million deaths and 129 million DALYs annually [1]. CAD comprises a broad spectrum of clinical disorders, ranging from asymptomatic atherosclerosis and stable angina to acute coronary syndrome [2]. Despite the fact that some notable advances have been made in the clinical management of CAD in recent decades, there are still many issues to address in order to limit the impact of this condition [3]. In this sense, the choice of the most appropriate available medical treatment for each patient has been at the forefront of different investigations, since there are still many questions to be answered [2]. 

Percutaneous coronary intervention (PCI) and coronary artery bypass grafting (CABG) have evolved from procedures in the experimental stage to hospital routine procedures that can safely be performed on high-risk patients and in complex coronary anatomic cases [4,5,6,7]. Both PCI and CABG are considered revascularization procedures, although the mechanistic action of each one is intrinsically different, which should be taken into account in clinical decision-making [8]. PCI has been proven to be the safest way of revascularization for acute myocardial infarction (MI) [9], being equivalent to CABG in terms of mortality for more stable patients with single- or multivessel disease and low surgical risk [4]. Thus, PCI tends to be more commonly used due to the less invasive nature of the technique [5]. However, CABG has been observed to present higher long-term benefits for certain subgroups of patients, both in terms of mortality rates and risk of re-intervention. For instance, when comparing CABG and PCI for multivessel disease and diabetic patients, CABG seems to hold lower mid–long-term all-cause mortality numbers [4,5,10]. Additionally, in recent years, many studies have observed an improvement in post-intervention mortality for patients undergoing CABG.

Historically, a significant decrease in CABG operations has been documented since the emergence of PCI. For instance, in OECD-12 (Organisation for Economic Co-operation and Development), CABG decreased from around 60% of total revascularization procedures in 1990 to less than 30% in 2007 (*HealthCare at a Glance*—OECD Indicators, 2009) [11,12,13,14]. However, due to the latest improvements in CABG and the emergent evidence on overall long-term outcomes in some patients in favor of CABG, this trend is far from homogeneous, and in recent years, some countries have started to perform more CABG, as it is the case for Iceland, Portugal, and the USA [15].

Likewise, we can observe large disparities in indications for PCI and CABG between hospitals and countries nowadays. As an example, in European countries [16], CABG was performed on 20.5% of eligible patients, but there were countries with rates of more than 30% for performing CABG (such as Poland, Finland, and Denmark), while in others the rate of performance was less than 15% (such as France and Spain). 

In this context, given the high heterogeneity in the use of both PCI and CABG, it is not easy to compare both procedures in terms of mortality and adequacy for each patient. However, it is more feasible to study how these two procedures have evolved over time. Thus, the aim of the present study is to analyze the intervention outcomes of patients that underwent PCI or CABG in Spain in two periods, namely between 2010 and 2012 and between 2016 and 2019, to assess the evolution of each procedure through recent years and how this evolution influenced the use of each procedure. 

## 2. Patients and Methods

The present study is designed as an observational, analytical, and retrospective cohort study. It utilizes a database that includes all hospital discharges from CABG and PCI procedures performed in Spain from 2010 to 2012 and from 2016 to 2019. The data, obtained from the Spanish Public Health Ministry, comprise 404,918 hospital discharges.

For each patient, two categories of information were provided: socio-demographic and medical. Socio-demographic data include age, gender, and the Spanish region of origin. Medical data cover the type of intervention (PCI or CABG), the date of the procedure, whether it was urgent or scheduled, the coded diagnoses the patient received (International classification of disease (ICD)-9-CM (CM = clinical modification) for the 2010–2012 period and ICD-10-ES (ES = Spanish) ( for the subsequent years), and whether the patient survived or died at the time of hospital discharge. Based on previous studies, three key comorbidities are associated with both procedure selection and mortality risk in CABG and PCI: diabetes mellitus [17], heart failure [18,19], and kidney diseases [20,21,22]. Therefore, the focus is on these three comorbidities.

The aim of this study is to analyze the evolution of mortality rates after PCI or CABG in the two time periods and assess how this evolution influenced the utilization of each procedure. To achieve this, the study tests whether the differences between the two periods are statistically significant. Mortality rates are then adjusted for socio-demographic and medical information to determine whether observed differences are due to variations in patient characteristics or medical performance. Two multivariate logistic regression models are used, one for PCI and one for CABG. In both models, the dependent variable is binary, indicating whether the patient died (coded as 1) or survived (coded as 0) following discharge. Covariates include age, gender, and binary indicators for diabetes, heart failure, kidney disease, whether the procedure was urgent or scheduled, and the time period. This last variable captures the significance of differences between the two periods after adjusting for other medical and demographic factors. If there was an improvement in performance, the parameter estimate would be negative (indicating a reduced mortality risk) and statistically significant.

To provide a more comprehensive analysis, a univariate examination of mortality risk by procedure and the other covariates is also conducted. This allows the observation of which patient subgroups experienced the most notable changes.

Finally, the study examines whether physicians incorporate recent survival data when recommending PCI or CABG. It explores how physicians balance two types of information: historical performance and current trends. During the second time period, physicians could rely on the survival gap between CABG and PCI from the first period (historical data) and the improvement CABG showed in the second period (current trends). The study tests whether, during the second period, CABG usage increased more significantly in patient segments where CABG had previously underperformed or improved the most.

The hypothesis is that physician decision-making is influenced by both past performance and current trends. Specifically, a lower CABG performance in the past is expected to result in reduced CABG usage, while greater improvements in past CABG performance are expected to increase its usage in the present.

To conduct this analysis, a model is developed to assign CABG versus PCI for each time period. These models consist of multivariate logistic regressions where the outcome variable is binary, indicating whether the patient received CABG (coded as 1) or PCI (coded as 0). The covariates used are the same as those in the mortality adjustment models. The models estimate the likelihood of a patient being assigned to CABG surgery based on medical and demographic conditions for each time period. The probabilities of being assigned CABG in the first period (2010–2012) and the second period (2016–2019) are noted as $p_{1}$ and $p_{2}$, respectively. Additionally, the probabilities of survival for PCI and CABG in each time period are represented as ${survPCI}_{1}$, ${survPCI}_{2},\;survCABG_{1}$, and ${survCABG}_{2}$, respectively. The study tests whether the difference in expected mortality rates between CABG and PCI for each patient in the first period (historical information:$\;{survCABG_{1}-survPCI}_{1}$) and the relative improvement in survival rates for CABG in the second period (current trends: ∆survCABG−∆survPCI) influences the change in the probability of being assigned CABG between the two periods ($p_{2}-p_{1}$).

## 3. Results

### 3.1. Sociodemographic Data of the Patients

We obtained data from 404,918 hospital discharges with PCI or CABG during the periods of study. Table 1 is a descriptive overview of the socio-demographic data of the hospital discharges involved in the study, by time period and procedure. We also include the main comorbidities associated with mortality risk, as well as the urgency of the procedures.

### 3.2. Evolution of the Mortality Rate of CABG 

The crude mortality rate for CABG was 5.6% in the 2010–2012 period and reduced to 4.8% in the 2016–2019 period (*p* < 0.001). As can be seen in Table 2, after adjusting mortality rates by gender, age, whether it was an urgent intervention or not, and comorbidities (heart failure, renal insufficiency, and diabetes mellitus), the improvement in mortality rates for CABG was consistent (*p* < 0.001). Hence, there is statistical evidence that CABG mortality significantly reduced in 2015–2019 with respect to 2010–2012. All in all, CABG mortality improved by 0.9%.

### 3.3. Evolution of Mortality Rate by Type of Patient in CABG 

**Figure 1:**
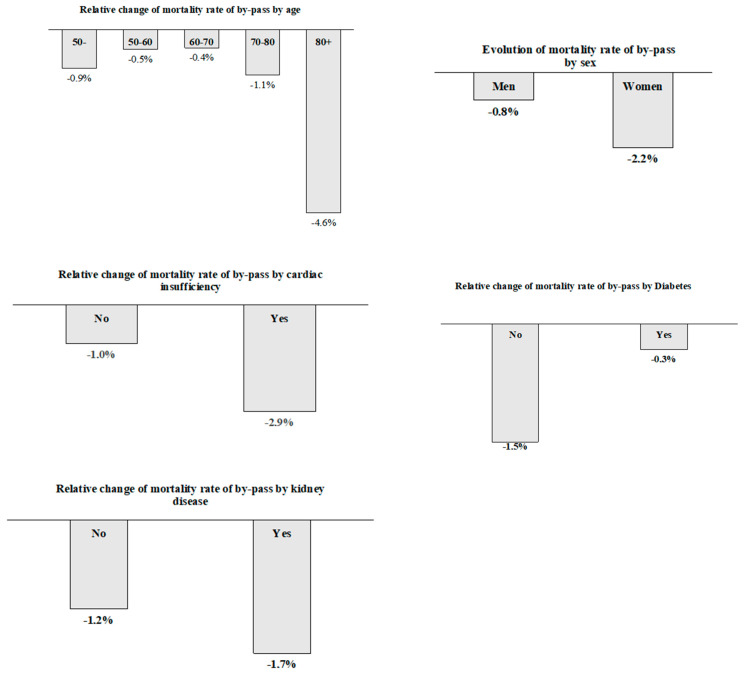
Relative change in mortality rate of CABG by variable.

The overall relative improvement in CABG during the two periods was not equal for all patient segments. As we can see in Figure 1, in terms of age, the eldest patients (older than 80 years) were the ones who most benefitted from the improvement. Indeed, the mortality rate of CABG was reduced by 4.6%. Regarding gender, mortality in women was reduced by 2.2% while, in men, it was decreased by 0.8%. In patients with heart failure, the mortality was reduced by 2.9%, while, in those not experiencing heart failure, it was reduced by 1%. Regarding diabetes, the reduction was higher for those without it, with a 1.5% reduction, versus 0.3% less mortality in those patients with diabetes. In patients with kidney disease, the trend was similar to that of patients with heart failure, with a greater reduction in those patients with said comorbidities. In summary, the evolution of the mortality rates for CABG was better (that is, with a higher reduction) for the most complex patients: women, 80+ years old, and those with multiple comorbidities.

### 3.4. Evolution of the Mortality Rate of PCI 

The crude mortality rate for PCI was 2.3% in the 2010–2012 period and increased to 2.7% in the 2016–2019 period (*p* < 0.001). The mortality rates were also adjusted by gender, age, whether it was an urgent intervention or not, and comorbidities (heart failure, renal insufficiency, and diabetes mellitus), with a *p*-value of 0.013. As can be seen in Table 3, the PCI mortality rates remained almost constant in both periods of time.

### 3.5. Evolution of Mortality Rate by Type of Patient in PCI 

**Figure 2:**
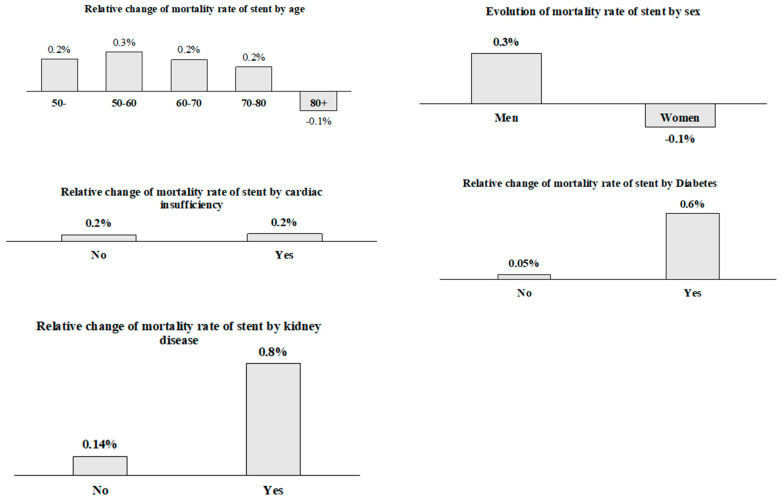
Relative change in mortality rate of PCI by variable.

As shown in Figure 2, the overall mortality rates for PCIs remained stable, with minimal shifts between both periods. The larger changes we observed involved a 0.6% mortality rate increase in diabetic patients and a 0.8% mortality rate increase in kidney disease patients.

### 3.6. Modification of the Indication of Procedure in the Second Period

Finally, we present the results of the information model, which measures how physicians incorporate the improvements in bypass survival rate into their decision regarding whether to recommend a CABG or a PCI. The results are presented in Table 4. As we can see, the higher the survival rate of the bypass (CABG), with respect to PCI, in the first period (the *initial survival difference* variable, which corresponds to the difference in survival rate between CABG and PCI during 2010–2012), the higher the likelihood that a patient is assigned to CABG in the second period. Moreover, the higher the improvement in CABG with respect to PCI, (the *survival difference change* variable, which corresponds to the difference in survival rate between CABG and PCI during the two periods), the higher the increase in the CABG assignment rate. Per each percentage point of increase in CABG survival with respect to PCI, the assignment rate of CABG increased by 0.628 points.

In summary, the results of our analysis show three conclusions. First, the mortality rate of CABG has experienced a significant reduction of 0.8 percentage points during recent years in Spain, which is consistent with the evidence from other countries. Second, this reduction is not due to a change in the patients’ characteristics but to improvements in the procedure. Indeed, the reduction in mortality rate for CABG is higher in more complex patients. For example, the mortality rate for patients aged 80+ years old has decreased by 4.6 percentage points, compared to a general reduction of 0.8 percentage points. Finally, our last conclusion is that physicians have incorporated the improvements in CABG survival rates into their decision regarding whether to recommend a bypass or a stent. Per each percentage point of increase in CABG survival with respect to PCI, the assignment rate of CABG increased by 0.628 points.

## 4. Discussion

### 4.1. Main Findings

In this work, we have evaluated the evolution of PCI and CABG in terms of mortality rate over two periods of time in Spain. We found that the overall mortality rate of PCI was markedly lower than that of CABG. However, when considering the evolution of both procedures over time, we observed that the CABG mortality rate displayed a marked reduction (from 5.6% in 2010–2012 to 4.8% in 2016–2019), whereas the PCI mortality rate demonstrated a slight increase (from 2.3 in 2010–2012 to 2.7% in 2016–2019). Remarkably, the improvement in the CABG mortality rate and the slight decrease in the PCI mortality rate has been achieved in a context of higher complexity, namely in elderly patients with more comorbidities (see Table 1). Overall, we observed a 1% reduction in the CABG mortality rate when compared with that of PCI (adjusted by age, gender, comorbidities, and type of intervention), and this percentage is even higher when compared with certain subgroups. These results agree with previous works, in which the CABG mortality rate has tended to decrease over time while the PCI mortality rate has remained almost the same [5,23]. Thus, the observations made in our study are similar to those obtained in previous works, although the interpretation of our results should be performed cautiously.

First, it should be noted that differences in the mortality rate between PCI and CABG could be related to differences in the number of patients undergoing each procedure. Previous works have observed an increase in the total number of patients receiving PCI, to the detriment of those receiving CABG in Spain over time [24]. The factors implicated in this fact can be quite diverse. For instance, Kim et al. [25] observed in Korea that the increased number of PCI procedures and the reduction in CABG procedures was directly related to a decreased number of cardiothoracic surgeons available and also directly associated with the number of hospital beds available. Shawon et al. [26] also reported, in Australia, a dramatic shift from using CABG to using PCI over time, with the patient base for PCI extending to older and sicker patients, which helped to explain the decreased proportion of the CABG mortality rate and the absence of variation after PCI. In our study, we also observed an increased proportion of patients with complications who had undergone PCI in the second period compared to the first, and the number of urgent cases increased from 72.7% in 2010–2012 to 78% in 2016–2019, whereas, for CABG, this increase was lower (from 36.8% to 39.3%). Likewise, the subgroups in which we found a larger CABG mortality rate reduction were also the ones where higher mortality rates had been reported in the past: elderly patients, diabetes, kidney disease, heart failure, and women. Despite observing improvement in the mortality rate of these subgroups, their mortality rate remained slightly higher than the mean. In the event of PCI, we only observed a moderate decrease over time of the mortality rate in women and people aged over 80 years old (both of which were 0.1%). Thus, and in agreement with a prior study [27], we believe that the differences in the clinical profiles of patients undergoing PCI and CABG over time can be directly associated with our observations, although further studies are still warranted in this sense. 

Another important endpoint of our study was to assess whether a change in the CABG mortality rate led to a consequential change in the indication for the procedure. We found a greater increase in the indication for CABG in those types of patients for whom the CABG mortality rate improved the most. Specifically, for every percentage point increase in survival for CABG over PCI, the CABG allocation increased by 0.628 points. These results are in line with the recent trend of an increase in the number of CABGs performed that has been observed in the US and in other countries in the OECD [15], although Spain remains at the head of the OECD in the use of PCI procedures, with respect to CABG. To our knowledge, there are no reports indicating longer survival attributed to a specific technique. However, as all data are standardized for patient characteristics and mortality, technical advancements such as minimally invasive surgery, robotic surgery, improved patient selection, and enhanced on-pump and off-pump techniques appear to be the factors contributing to the improvement in the CABG mortality rate.

To our knowledge, this study is the first to measure the efficiency and speed at which new scientific evidence is integrated into daily clinical practice. The absence of similar studies in other medical treatments and procedures makes it challenging to establish a benchmark to determine whether the pace of evidence adoption in PCI and CABG is optimal. To the best of our knowledge, there is no previous literature on the evolution of CABG and PCI in the Spanish population, adjusted by patients’ characteristics. No previous paper found that the mortality rate reduction was more relevant in complex patients, and no other paper analyzed whether and at which rate physicians were re-adjusting their procedure selection (PCI or GABC) according to expected outcomes, by patient characteristics. These have several clinical implications, as follows: (1) improvements in procedures require continuous monitoring to ensure optimal patient management. Even in well stablished procedures, such as PCI and CABG, technical advancements can have a significant impact on their outcomes that may require physicians to adjust the clinical criteria; (2) although overall results are relevant, segmented analysis is key to optimal decision-making. For example, CABG for 80+ year old patients demonstrated an extraordinary improvement in survival rates. Conversely, PCI may have a worsened performance for patients with diabetes or kidney disease, which could require further research to understand if there are underlying causes that could be addressed; (3) knowledge spread among physicians is also key to optimal decision-making. Although we cannot assess whether the velocity estimated in our analysis (0.628 percentage points in shifting towards “improving” the technique, for each percentage point of relative improvement in the survival rate) is good or not, our paper can serve as an initial benchmark for future analysis on knowledge spread.

Future research should aim to extend this type of measurement to other areas of medicine, thereby establishing a reference benchmark. While improvements in CABG outcomes have been observed across all age groups, they are particularly notable in patients over 80 years old. Although these findings are promising from a procedural and technical perspective, further research should explore the broader social impact, particularly in terms of the DALYs and Quality Adjusted Life Year (QALYs) gained through the allocation of these resources.

### 4.2. Limitations 

The major limitation of our study was the absence of data in our database that could help the study to achieve more accurate results, as it is relevant in the prognosis of CAD patients undergoing PCI and CABG. Such non-available data, which have been repeatedly studied in the literature, include long-term mortality, lack of coronary anatomic information [28], body mass index [29,30,31], or smoking habits [32,33,34]. For instance, international scientifically proven scales to assess the risk for patients undergoing PCI or CABG, such as SYNTAX score, EuroScore or The American College of Cardiology (ACC)/American Heart Association (AHA) score, could not be implemented in this cohort. Furthermore, since the only endpoint of our study was mortality rate, this paper does not control the procedure results by the emergence of important complications such as myocardial infarction or stroke. All these non-observed data are crucial in the assignation and management of coronary disease. 

## 5. Conclusions

Although PCI remains the most prevalent technique for coronary revascularization and the procedure with a lower mortality rate, recent data show a catch-up in CABG post-procedure survival rates, with respect to PCI results, in conjunction with better results for CABG in the long term for DM patients or those experiencing multivessel disease, and a lower rate of mid–long-term complications. Despite the heterogeneity in CABG and PCI indications among different countries, we observed a mild increase in CABG indication, especially in the specific cases mentioned previously. 

## Figures and Tables

**Table 1:** Description of the data.

	PCI		CABG	
	2010–12	2016–19	2010–12	2016–19
Age	65.4	66.1	67.4	67.2
	(12.3)	(12.3)	(10.1)	(10.0)
Women	23.3%	23.4%	21.4%	19.8%
Diabetes	31.9%	33.2%	36.5%	41.1%
Kidney disease	6.8%	9.3%	7.5%	10.7%
Heart failure	11.0%	11.9%	11.1%	12.6%
Urgent intervention	72.7%	78.0%	36.8%	39.3%
Mortality rate	2.3%	2.7%	5.6%	4.8%
Number of discharges	154.749	203.111	20.067	26.991

Note: Differences in % of women, diabetes, kidney disease, heart failure, urgent intervention, and mortality rate are statistically significant at <0.01 (mean difference *t*-test).

**Table 2:** Adjusted and unadjusted mortality rates.

	Mortality Rate				Adjusted Mortality Rate			
	2010–12	2016–19	Dif.	*p* Value	2010–12	2016–19	Dif.	*p* Value
CABG	5.6%	4.8%	−0.8%	<0.001	5.6%	4.7%	−0.9%	<0.001

**Table 3:** Adjusted and unadjusted mortality rates.

	Mortality Rate				Adjusted Mortality Rate			
	2010–12	2016–19	Dif.	*p* Value	2010–12	2016–19	Dif.	*p* Value
PCI	2.3%	2.7%	0.4%	<0.001	2.3%	2.5%	0.1%	0.013

**Table 4:** Estimated effects of prior and posterior information about mortality rates on changes in CABG assignation.

Effects	Estimates
Constant	0.0279 *
	(0.0001)
Initial survival difference (prior)	0.2902 *
	(0.0036)
Survival difference change (posterior)	0.6283 *
	(0.0101)
Number of observations	236,632
R-squared	0.0375

Significance: * *p* value < 0.001.

## Data Availability

The data used to support the findings of the present study are available from the corresponding authors upon request.
